# Impaired sensitivity to thyroid hormones is positively associated to metabolic syndrome severity in euthyroid Chinese adults as revealed by a cross-sectional study

**DOI:** 10.3389/fendo.2025.1552484

**Published:** 2025-05-15

**Authors:** Xiong Zhou, Ye Zhang, Zengyao Li

**Affiliations:** Department of Minimally Invasive Laparoscopy, The Affiliated Wuxi People’s Hospital of Nanjing Medical University, Wuxi People’s Hospital, Wuxi Medical Center, Nanjing Medical University, Wuxi, China

**Keywords:** thyroid hormones sensitivity, metabolic syndrome, severity, euthyroid, Chinese adults

## Abstract

**Objective:**

Thyroid hormones (THs) play a pivotal role in regulating metabolism, and their sensitivity may influence the risk of metabolic syndrome (MetS). This study aimed to investigate the association of impaired sensitivity to THs with MetS and MetS severity score (MetSSS) in Chinese euthyroid adults.

**Methods:**

A cross-sectional analysis was conducted involving 17,272 health check-up participants. THs sensitivity indices, including Thyroid Feedback Quantile-Based Index (TFQI), Parametric Thyroid Feedback Quantile-Based Index (PTFQI), TSH Index (TSHI), Thyrotropin Thyroxine Resistance Index (TT4RI), and free triiodothyronine/free thyroxine (FT3/FT4) ratio were assessed. Multivariable regression and restricted spline cubic analyses were conducted to explore the association between THs sensitivity indices and MetS and MetSSS. Subgroup analysis was also performed to examine this association stratified by sex and age.

**Results:**

Multivariable logistic regression analysis indicated that MetS risk was positively associated with all impaired THs sensitivity indices (per SD increase) (TFQI: OR=1.20, 95%CI: 1.15-1.25); PTFQI: OR=1.28, 95%CI: 1.23-1.33; TSHI: OR=1.35, 95%CI: 1.29-1.42; TT4RI: OR=1.57, 95%CI: 1.47-1.67; FT3/FT4: OR=1.17, 95%CI: 1.12-1.23)(all P-value<0.001). After adjusting for confounders, compared with the lowest group of MetSSS, individuals in the highest group of MetSSS were positively associated with all impaired THs sensitivity indices (per SD increase) (TFQI: OR=1.16, 95%CI: 1.07-1.21 PTFQI: OR=1.12, 95%CI: 1.06-1.17; TSHI: OR=1.13, 95%CI: 1.06-1.19; TT4RI: OR=1.25, 95%CI: 1.15-1.35; FT3/FT4: OR=1.82, 95%CI: 1.72-1.93). Nonlinear associations were found between THs sensitivity indicators and MetS (P for non-linear<0.001). Subgroup analysis indicated that all thyroid hormones sensitivity indices were positively associated with MetS by gender (male/female) and age (<60 years/≥60 years).

**Conclusion:**

Impaired sensitivity to THs is associated with an increased risk of MetS and MetSSS in Chinese euthyroid adults. Future research should consider thyroid hormones sensitivity indices in the assessment of MetS risk.

## Introduction

Metabolic syndrome (MetS) is a complex metabolic disorder characterized by the presence of central obesity, high blood sugar levels, abnormal lipid levels, and high blood pressure (1). Central obesity, with excess visceral fat, affects insulin sensitivity and vascular health. Elevated blood sugar indicates insulin resistance, leading to atherosclerosis and microvascular damage. Dyslipidemia increases plaque formation risk, while hypertension, tied to insulin resistance and obesity, worsens vascular issues. Collectively, these components boost the risk of cardiovascular diseases and type 2 diabetes (2). The prevalence of MetS has become a major global health concern, with the International Diabetes Federation estimating that one in four people worldwide are affected (2, 3). In China, the situation is equally troubling, as there has been a noticeable rise in the prevalence of MetS in recent years, highlighting the need for further research in this area (4).

Thyroid hormones (THs) are crucial for regulating development, metabolism, homeostasis in multiple organ systems and various physiological functions including energy balance. The two primary thyroid hormones are thyroxine (T4) and triiodothyronine (T3). T4, mainly secreted by the thyroid, acts as a prohormone, while T3, generated via tissue-specific deiodination of T4, is the biologically active form that binds to nuclear thyroid hormone receptors (TRs) to regulate gene transcription (genomic effects) (5).

Emerging evidence highlights the significance of thyroid hormone metabolites, which were once considered inactive byproducts. These metabolites include reverse T3 (rT3), tetraiodothyroacetic acid (Tetrac), triiodothyroacetic acid (Triac), diiodothyronines (e.g., 3,5-T2 and 3,3’-T2), and thyronamines (e.g., 3-T1AM). They exhibit distinct biological activities, particularly through non-genomic pathways. For example, 3,5-T2 modulates mitochondrial energy expenditure and lipid metabolism, while 3-T1AM influences thermoregulation and neuronal signaling. These metabolites often function independently of classical TR-mediated mechanisms, playing roles in fine-tuning physiological processes such as metabolic rate, cardiovascular function, and central nervous system activity (6). Both hypothyroidism and hyperthyroidism can result in insulin resistance and have a detrimental impact on glucose and lipid metabolism, thereby being associated with the development of MetS (7, 8). Understanding the diversity and functional interplay of thyroid hormones and their metabolites is essential for elucidating their contributions to health and disease, particularly in metabolic disorders where traditional TH signaling may be dysregulated.

The study’s biochemical basis is the complex link between thyroid hormones and metabolism. Thyroid hormones are key to energy balance, glucose and lipid metabolism, and cardiovascular function, with both genomic and non-genomic effects (9). Genomically, they bind to nuclear receptors, affecting genes related to glucose and lipid metabolism, which impacts insulin sensitivity and lipid profiles (10). For example, they boost insulin secretion and receptor expression. Non-genomically, thyroid hormones interact with cell membranes and cytoplasmic proteins, rapidly modulating ion channels and enzymes involved in energy and glucose metabolism (11). They also stimulate mitochondrial biogenesis and uncoupling proteins, influencing energy use and lipid oxidation. Moreover, thyroid hormones interact with other hormones like insulin and adrenaline, affecting metabolic regulation. The hypothalamus-pituitary-thyroid feedback loop controls thyroid hormone levels, and its disruption can cause metabolic issues (12).

Previous research has shown conflicting results when it comes to the relationship between thyroid function and MetS. Some studies have suggested a connection between normal levels of thyroid-stimulating hormone (TSH) and the presence of MetS (13, 14). while others have not found any association or have pointed to FT4 instead (15, 16). These discrepancies could be due to differences in study populations, methodologies, and the failure to consider potential confounding factors like gender, which has been shown to impact the prevalence of MetS components and their correlation with THs (17–19). Additionally, simply measuring TSH, FT3, and FT4 levels in individuals with normal thyroid function may not be enough to accurately assess thyroid function status. It is important to recognize that thyroid hormone balance may not be stable even if these markers fall within the normal range.

Recently, there has been a growing interest in the idea that reduced sensitivity to THs in the general population could play a role in metabolic disorders (20). This concept of THs sensitivity considers both FT4 and TSH levels. In cases of THs resistance syndrome, elevated levels of both FT4 and TSH are present, indicating issues with energy regulation. Normally, there is a negative correlation between THs and TSH due to the feedback loop of the hypothalamic-pituitary-thyroid axis (21, 22). However, individuals with mild resistance to THs may have high levels of both hormones. Researchers have developed various indices, such as the thyroid feedback quantile-based index (TFQI), parametric thyroid feedback quantile-based index (PTFQI) (20), thyrotrophic thyroxine resistance index (TT4RI) (23), and thyroid-stimulating hormone index (TSHI) (24), to quantify the relationship between thyroid function and metabolic factors. These indices help to clarify conflicting findings regarding the link between THs and MetS.

Previous studies have demonstrated a direct link between sensitivity to THs and certain health issues like prediabetes, decreased kidney function, and higher risk of cardiovascular disease (25–27). Yet, there has been a lack of investigation into the relationship between THs sensitivity and MetS in individuals with normal thyroid function. This cross-sectional study seeks to investigate the link between sensitivity to THs and MetS, as well as its severity score, in a sizable group of Chinese euthyroid adults. Through the evaluation of various THs sensitivity indices, we aim to gain a more detailed understanding of the association between sensitivity to THs and MetS in euthyroid Chinese adults. The results could shed light on potential risk factors for MetS and aid in the creation of personalized prevention and treatment plans for Chinese adults.

## Materials and methods

### Study population and design

The study included adults over 18 years old who had undergone annual health examinations at the health check-up center of People’s Hospital in Wuxi city, affiliated with Nanjing Medical University. Initially, a total of 25,360 individuals were part of this retrospective study. Participants were excluded if they had incomplete medical information, lacked blood parameters for thyroid function tests, had a history of thyroid surgery or were taking thyroid medication, were not euthyroid, or had oncology, severe liver, or kidney dysfunction. After excluding these individuals, the study included 17,272 participants, comprising 10,442 males and 6,830 females aged 18 to 89 years. This retrospective study was approved by the Health Examination Center of People’s Hospital in Wuxi city (approval number: not applicable), affiliated with Nanjing Medical University, following the principles of the Declaration of Helsinki. Patient data was anonymized to ensure confidentiality, and statistical analysis was conducted securely for scientific research purposes. Therefore, informed consent was waived.

### Data collection

We used a standard questionnaire to gather information on participants’ age, gender, and use of cigarettes and alcohol. Smoking was defined as consuming three or more cigarettes daily for a year, while alcohol consumption was defined as drinking at least three times a week for twelve months. Participants provided fasting venous blood samples after a 12-hour overnight fast. Levels of fasting plasma glucose (FPG), triglycerides (TG), total cholesterol (TC), high-density lipoprotein cholesterol (HDL-C), low-density lipoprotein cholesterol (LDL-C), neutrophils (NE), and lymphocytes (LY) were measured using an automatic hematology analyzer. The neutrophil to lymphocyte ratio (NLR) was calculated. Strict quality control procedures were followed in the laboratory.

In addition, we gathered information on individuals’ health, such as whether they had been previously diagnosed with hypertension or diabetes, and if they were currently taking any medications. Diabetes was defined as having fasting blood glucose levels of 7.0 mmol/L or higher, being prescribed insulin or oral hypoglycemic agents, or self-reporting a history of the condition (28). Hypertension was determined by having a systolic blood pressure of 140 mmHg or higher, or a diastolic blood pressure of 90 mmHg or higher, and currently using antihypertensive medications (29). We used the electrochemiluminescence immunoassay method to measure the concentrations of thyroid-stimulating hormone (TSH), free triiodothyronine (FT3), and free thyroxine (FT4). The reference ranges for FT3, FT4, and TSH were 3.10 to 6.80 pmol/L, 12.00 to 22.00 pmol/L, and 0.27 to 4.20 mIU/L, respectively. Euthyroid was defined as having serum TSH and FT4 levels within the normal ranges and not using thyroid hormone medication.

The physical examination included measuring height (in centimeters), weight (in kilograms), waist circumference (in centimeters), and blood pressure (in mmHg). BMI was calculated by dividing weight in kilograms by height in meters squared. Systolic and diastolic blood pressure were measured on the right arm using a sphygmomanometer after at least 5 minutes of rest, and the average of two readings was recorded.

### Metabolic syndrome and MetS severity score

Metabolic syndrome (MetS) is defined according to the 2009 guidelines of the International Diabetes Federation (IDF) and the American Heart Association/National Heart, Lung, and Blood Institute (AHA/NHLBI) (30, 31). It includes three of the following five criteria: 1) elevated waist circumference, with specific measurements for men (≥90 cm) and women (≥80 cm); 2) elevated triglycerides (≥150 mg/dl) or use of medication for high triglyceride levels; 3) low HDL cholesterol (<40 mg/dl in men and <50 mg/dl in women) or use of medication for low HDL levels; 4) high blood pressure (systolic ≥130 mmHg and/or diastolic ≥85 mmHg) or use of antihypertensive medication; and 5) elevated fasting glucose (≥100 mg/dl) or use of medication for high glucose levels. Additionally, a Metabolic Syndrome Severity Score (MetSSS) is calculated based on specific equations for age, sex, and ethnicity (**Table 1**) (32).

**Table 1:** Age-sex-ethnicity-specific MetSSS equations.

Groups	MetSSS equations
Male	
<60 years	-2.9092 + 0.0262*WC +0.3098*TG-0.944*HDL-C+0.0097*MAP+0.0745*FBG
≥60 years	-2.3741 + 0.0264*WC+0.4933*TG-0.999*HDL-C+0.0054*MAP+0.0821*FBG
Female	
<60 years	-2.4981 + 0.0199*WC+0.5218*TG-0.8616*HDL-C+0.0110*MAP+0.1074*FBG
≥60 years	-0.5682 + 0.0153*WC+0.4587*TG-1.3567*HDL-C+0.0036*MAP+0.0688*FBG

### Indices of thyroid hormone sensitivity

The participants’ central sensitivity to THs was assessed using Thyroid Feedback Quartile-Based index (TFQI), parametric thyroid feedback quantile-based index (PTFQI), TSH index (TSHI), and Thyrotroph T4 Resistance Index (TT4RI). Higher values of TFQI, PTFQI, TSHI, and TT4RI indicate lower central sensitivity to thyroid hormones. Peripheral THs sensitivity was evaluated using the FT3 to FT4 ratio (FT3/FT4), where higher values suggest higher sensitivity. The equations for calculation are provided with the following formulas (16, 19, 20): TFQI = cdf FT4 − (1 − cdf TSH); cdf: cumulative distribution function. PTFQI = φ((FT4-μFT4)/σFT4) - (1-φ ((ln TSH-μln TSH)/σlnTSH)), where μfT4 = 15.70, σfT4 = 1.80, μln TSH=0.62, and σln TSH=0.45 for the Chinese population. TSHI = ln TSH (mIU/L) + 0.1345 × FT4 (pmol/L). TT4RI = FT4 (pmol/L) × TSH (mIU/L). FT3/FT4 = FT3 (pmol/L)/FT4 (pmol/L).

### Statistical analyses

The statistical analyses were conducted using SPSS 26.0 (Chicago, IL, USA) and R software (version 4.1). The normality of the variables was assessed using the Kolmogorov–Smirnov test. Normally distributed variables are presented as mean (standard deviation), skewed variables as median [interquartile range], and categorical variables as frequencies (proportions). One-way ANOVA test or Kruskal-Wallis H test were used to compare continuous variables, while the chi-square test was used for categorical variables. Multivariable logistic regression analysis was performed to assess the associations between MetS risk and thyroid hormone sensitivity indices, adjusting for potential confounding factors. Two models were used for adjustment: Model 1 included sex and age, while Model 2 included variables from Model 1 as well as smoking, drinking, BMI, and NLR. The relationship between thyroid hormone sensitivity indices and the risk of MetS and MetSSS in all euthyroid participants was examined using restricted cubic spline analysis. The model incorporated 4 knots placed at the 5th, 35th, 65th, and 95th percentiles of SII, with the p-value indicating the nonlinearity of the smooth curve fitting. Subgroup analysis was conducted to explore the correlation between thyroid hormone sensitivity indices and the risk of MetS and its components among different subgroups based on sex (males/females) and age (≥60 years/<60 years). A significance level of P < 0.05 (2-tailed) was considered statistically significant.

## Results

### Baseline characteristics

**Figure 1:**
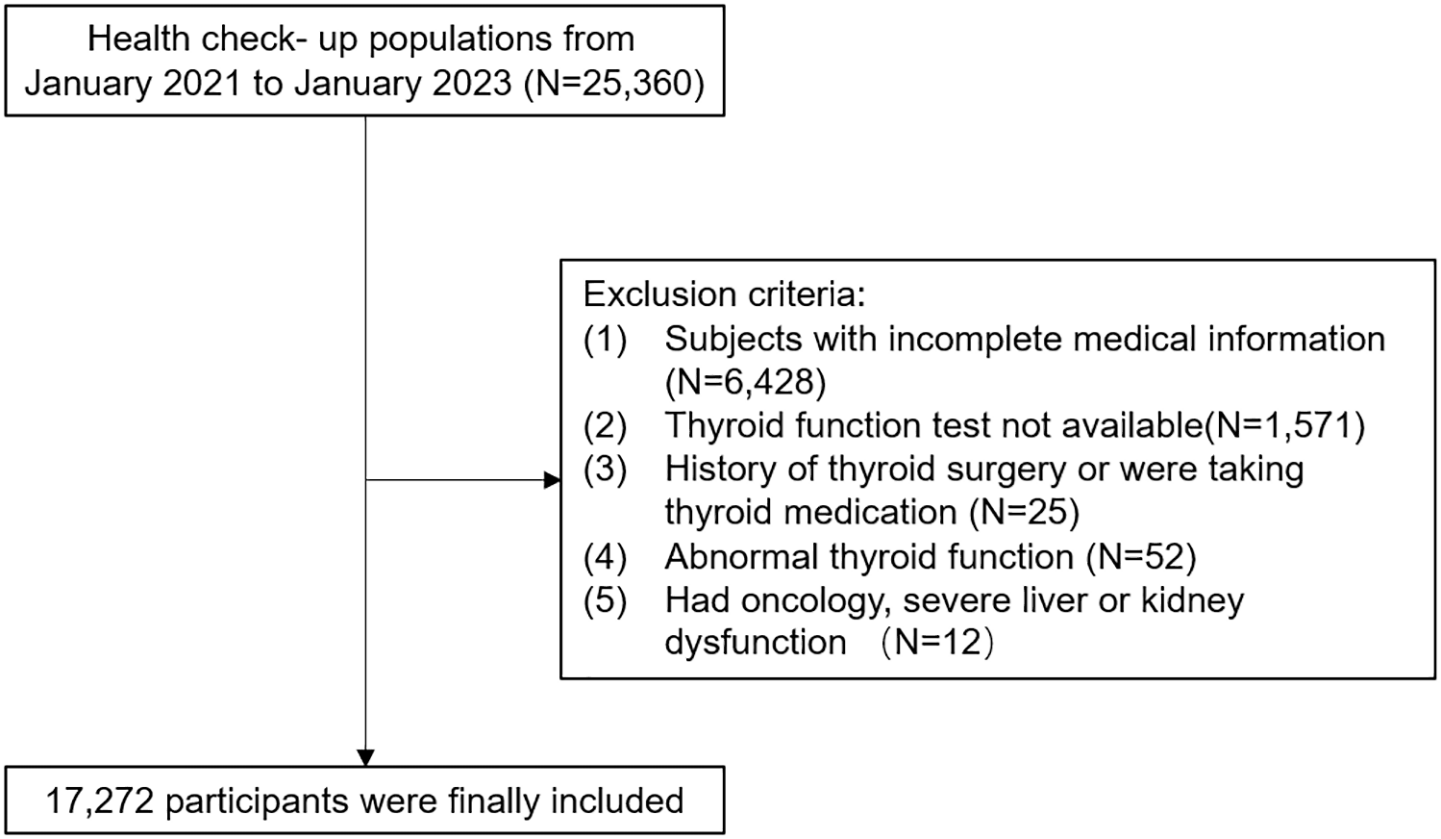
Study design flowchart.

**Table 2** presents the baseline characteristics of all participants categorized by quartiles of MetSSS. A total of 17,272 participants were included in the analysis, with 10,442 males (60.5%) and 6,830 females (39.5%) (**Figure 1**). There were significant differences in all baseline characteristics among the four quartiles of MetSSS (all P-values < 0.001). Individuals in the higher quartiles of MetSSS were more likely to be older, male, smokers, drinkers, and had higher rates of hypertension and diabetes (P for trend < 0.001). There were notable increases in BMI, WC, FBG, SBP, DBP, TG, TC, LDL-C, NLR, FT3, and FT4 across the quartiles of MetSSS, while HDLC decreased. Furthermore, participants in the higher quartiles of MetSSS exhibited higher levels of TFQI, PTFQI, TSHI, TT4RI, and FT3/FT4 (P for trend < 0.001).

**Table 2:** Baseline characteristics of all participants.

Characteristic	MetSSS quartile					P-value	P for trend
	Overall N = 17,272	Q1 N = 4,347	Q2 N = 4,341	Q3 N = 4,327	Q4 N = 4,257		
Age, years	50.64 ± 10.03	47.06 ± 10.55	50.26 ± 9.67	52.08 ± 9.12	53.21 ± 9.63	<0.001	<0.001
Male, n (%)	10,442 (60.5%)	1,532 (35.2%)	2,529 (58.3%)	3,086 (71.3%)	3,295 (77.4%)	<0.001	<0.001
Smoking, n (%)	4,892 (28.3%)	538 (12.4%)	1,033 (23.8%)	1,493 (34.5%)	1,828 (42.9%)	<0.001	<0.001
Drinking, n (%)	3,008 (17.4%)	381 (8.8%)	690 (15.9%)	884 (20.4%)	1,053 (24.7%)	<0.001	<0.001
BMI, kg/m2	24.5 ± 3.2	21.8 ± 2.3	23.9 ± 2.3	25.4 ± 2.5	27.1 ± 3.2	<0.001	<0.001
WC, cm	82.64 ± 9.74	73.61 ± 6.84	80.78 ± 7.02	85.76 ± 7.21	90.60 ± 8.60	<0.001	<0.001
FBG, mmol/L	5.52 ± 1.15	5.02 ± 0.48	5.30 ± 0.67	5.54 ± 0.93	6.22 ± 1.74	<0.001	<0.001
SBP, mmHg	121.73 ± 16.30	111.69 ± 14.04	119.32 ± 14.18	125.03 ± 14.64	131.08 ± 15.70	<0.001	<0.001
DBP, mmHg	73.78 ± 10.68	67.23 ± 8.86	72.25 ± 9.24	76.00 ± 9.73	79.76 ± 10.62	<0.001	<0.001
TG, mmol/L	1.27 (0.90, 1.87)	0.78 (0.64, 0.96)	1.10 (0.89, 1.35)	1.50 (1.21, 1.84)	2.39 (1.82, 3.24)	<0.001	<0.001
TC, mmol/L	4.86 (4.28, 5.46)	4.85 (4.30, 5.44)	4.80 (4.23, 5.38)	4.85 (4.26, 5.45)	4.94 (4.36, 5.61)	<0.001	<0.001
LDLC, mmol/L	3.14 (2.61, 3.68)	2.95 (2.49, 3.46)	3.17 (2.66, 3.71)	3.29 (2.76, 3.80)	3.16 (2.54, 3.72)	<0.001	<0.001
HDLC, mmol/L	1.28 (1.06, 1.54)	1.71 (1.53, 1.93)	1.35 (1.22, 1.51)	1.16 (1.04, 1.30)	1.00 (0.88, 1.13)	<0.001	<0.001
Diabetes, n (%)	1,221 (7.1%)	31 (0.7%)	128 (2.9%)	254 (5.9%)	808 (19.0%)	<0.001	<0.001
Hypertension, n (%)	2,201 (12.7%)	138 (3.2%)	333 (7.7%)	623 (14.4%)	1,107 (26.0%)	<0.001	<0.001
NLR	1.53 (1.21, 1.93)	1.44 (1.14, 1.85)	1.55 (1.21, 1.92)	1.55 (1.23, 1.95)	1.58 (1.27, 1.98)	<0.001	<0.001
FT3, pmol/L	4.70 (4.34, 5.08)	4.44 (4.12, 4.79)	4.69 (4.34, 5.03)	4.82 (4.47, 5.17)	4.86 (4.50, 5.22)	<0.001	<0.001
FT4, pmol/L	15.50 (14.40, 16.80)	15.30 (14.30, 16.30)	15.53 (14.50, 16.90)	15.57 (14.60, 17.00)	15.60 (14.40, 16.90)	<0.001	<0.001
TSH, mIU/L	1.92 (1.40, 2.58)	1.93 (1.40, 2.60)	1.89 (1.38, 2.57)	1.89 (1.38, 2.55)	1.96 (1.44, 2.63)	<0.001	0.154
TSHI	2.75 (2.43, 3.07)	2.73 (2.40, 3.04)	2.75 (2.42, 3.07)	2.75 (2.43, 3.06)	2.79 (2.46, 3.08)	<0.001	<0.001
TT4RI	30 (22, 40)	29 (21, 40)	29 (22, 38)	30 (22, 40)	32 (22, 41)	<0.001	0.001
PTFQI	-0.03 (-0.25, 0.18)	-0.06 (-0.28, 0.14)	-0.03 (-0.26, 0.18)	-0.02 (-0.25, 0.19)	0.00 (-0.22, 0.22)	<0.001	<0.001
TFQI	-0.07 (-0.27, 0.15)	-0.10 (-0.29, 0.10)	-0.06 (-0.26, 0.16)	-0.05 (-0.25, 0.16)	-0.05 (-0.26, 0.17)	<0.001	<0.001
FT3/FT4	0.30 ± 0.04	0.29 ± 0.04	0.30 ± 0.04	0.32 ± 0.03	0.34 ± 0.04	<0.001	<0.001

Continuous variables are presented as mean ± standard deviation or median (interquartile) with number (proportion, %) for categorical variables. P values among groups are calculated by one-way ANOVA or Kruskal-Wallis H tests for continuous variables, Chi-square test for categorical variables.

BMI, body mass index; WC, waist circumference; SBP, systolic blood pressure; DBP, diastolic blood pressure; FPG, fasting plasma glucose; TG, triglycerides; TC, total cholesterol; HDL-C, high-density lipoprotein cholesterol; LDL-C, low-density lipoprotein cholesterol; NLR, neutrophil to lymphocyte ratio; FT3, free triiodothyronine; FT4, free thyroxine; TSH, thyroid-stimulating hormone; TFQI, thyroid feedback quantile-based index; PTFQI, parametric thyroid feedback quantile-based index; TSHI, TSH index; TT4RI, thyrotropin thyroxine resistance index; FT3/FT4, free triiodothyronine to free thyroxine ratio.

### Adjusted odds ratios for sensitivity to THs and risk of MetS and its components

**Table 3** displays the adjusted odds ratios (ORs) and 95% confidence intervals (CIs) for the relationship between sensitivity to THs and the risk of MetS and its components. The analysis involved three models, including a crude model and two adjusted models controlling for various confounding factors. After adjusting for potential confounders such as age, sex, smoking, drinking, BMI, and NLR, the risk of MetS was found to be positively correlated with all sensitivity to THs [TFQI (+ 1 SD): OR=1.20, 95%CI: 1.15-1.25; PTFQI (+ 1 SD): OR=1.28, 95%CI: 1.23-1.33; TSHI (+ 1 SD): OR=1.35, 95%CI: 1.29-1.42; TT4RI (+ 1 SD): OR=1.57, 95%CI: 1.47-1.67; FT3/FT4 (+ 1 SD): OR=1.17, 95%CI: 1.12-1.23] (all P-value <0.001). Regarding MetS components, elevated waist circumference (WC) and low high-density lipoprotein cholesterol (HDL-C) did not show a significant association with thyroid sensitivity indices in the adjusted models. However, elevated blood pressure (BP), elevated triglycerides (TG), and elevated fasting plasma glucose (FPG) were positively associated with thyroid hormones sensitivity indices, with the strongest associations observed for FT3/FT4.

**Table 3:** Adjusted odds ratio (95% confidence interval) of sensitivity to THs and risk of MetS and its components.

	Crude model			Model 1			Model 2		
	OR	95%CI	P-value	OR	95%CI	P-value	OR	95%CI	P-value
MetS									
TFQI (+1 SD)	1.34	1.29, 1.40	<0.001	1.21	1.16, 1.26	<0.001	1.20	1.15, 1.25	<0.001
PTFQI (+1 SD)	1.35	1.30, 1.41	<0.001	1.26	1.21, 1.31	<0.001	1.28	1.23, 1.33	<0.001
TSHI (+1 SD)	1.37	1.30, 1.43	<0.001	1.34	1.27, 1.40	<0.001	1.35	1.29, 1.42	<0.001
TT4RI (+1 SD)	1.44	1.36, 1.53	<0.001	1.52	1.43, 1.62	<0.001	1.57	1.47, 1.67	<0.001
FT3/FT4 (+1 SD)	1.28	1.23, 1.34	<0.001	1.17	1.12, 1.22	<0.001	1.17	1.12, 1.23	<0.001
Elevated WC									
TFQI (+1 SD)	1.08	1.04, 1.11	<0.001	0.99	0.96, 1.03	0.710	0.99	0.95, 1.02	0.481
PTFQI (+1 SD)	1.07	1.04, 1.11	<0.001	1.03	0.99, 1.06	0.107	1.03	1.00, 1.07	0.059
TSHI (+1 SD)	1.05	1.01, 1.09	0.016	1.02	0.98, 1.06	0.319	1.02	0.98, 1.07	0.269
TT4RI (+1 SD)	1.05	0.99, 1.11	0.083	1.06	1.01, 1.12	0.045	1.07	1.01, 1.13	0.024
FT3/FT4 (+1 SD)	1.30	1.25, 1.35	<0.001	1.30	1.25, 1.36	<0.001	1.31	1.26, 1.36	<0.001
Elevated BP									
TFQI (+1 SD)	1.25	1.20, 1.29	<0.001	1.11	1.07, 1.15	<0.001	1.11	1.07, 1.15	<0.001
PTFQI (+1 SD)	1.13	1.10, 1.17	<0.001	1.06	1.02, 1.10	0.003	1.06	1.02, 1.10	0.002
TSHI (+1 SD)	1.15	1.10, 1.20	<0.001	1.11	1.06, 1.16	<0.001	1.11	1.07, 1.16	<0.001
TT4RI (+1 SD)	1.08	1.02, 1.14	0.005	1.11	1.05, 1.17	<0.001	1.12	1.05, 1.18	<0.001
FT3/FT4 (+1 SD)	1.26	1.21, 1.31	<0.001	1.20	1.15, 1.25	<0.001	1.20	1.15, 1.25	<0.001
Low HDL-C									
TFQI (+1 SD)	1.03	0.98, 1.09	0.269	1.12	1.04, 1.18	<0.001	1.15	1.08, 1.21	<0.001
PTFQI (+1 SD)	1.14	1.08, 1.20	<0.001	1.03	0.98, 1.09	0.213	1.06	1.01, 1.11	0.049
TSHI (+1 SD)	1.03	0.96, 1.09	0.418	0.99	0.93, 1.06	0.822	1.11	1.02, 1.16	<0.001
TT4RI (+1 SD)	1.00	0.92, 1.09	0.934	1.05	0.96, 1.14	0.288	1.10	1.01, 1.20	0.042
FT3/FT4 (+1 SD)	1.45	1.37, 1.53	<0.001	1.22	1.15, 1.29	<0.001	1.21	1.14, 1.29	<0.001
Elevated TG									
TFQI (+1 SD)	1.08	1.05, 1.12	<0.001	0.97	0.94, 1.01	0.098	1.12	1.04,1.18	<0.001
PTFQI (+1 SD)	1.16	1.12, 1.20	<0.001	1.07	1.03, 1.11	<0.001	1.08	1.04, 1.12	<0.001
TSHI (+1 SD)	1.10	1.06, 1.15	<0.001	1.08	1.03, 1.13	<0.001	1.09	1.04, 1.13	<0.001
TT4RI (+1 SD)	1.11	1.05, 1.17	<0.001	1.17	1.10, 1.23	<0.001	1.19	1.12, 1.26	<0.001
FT3/FT4 (+1 SD)	1.47	1.41, 1.53	<0.001	1.27	1.21, 1.32	<0.001	1.27	1.22, 1.33	<0.001
Elevated FPG									
TFQI (+1 SD)	1.25	1.21, 1.30	<0.001	1.11	1.07, 1.15	<0.001	1.10	1.06, 1.15	<0.001
PTFQI (+1 SD)	1.09	1.06, 1.13	<0.001	1.01	0.98, 1.05	0.492	1.09	1.03, 1.15	<0.001
TSHI (+1 SD)	1.12	1.07, 1.16	<0.001	1.07	1.03, 1.12	0.002	1.08	1.03, 1.13	<0.001
TT4RI (+1 SD)	1.04	0.98, 1.10	0.221	1.05	0.99, 1.12	0.092	1.07	1.01, 1.14	0.027
FT3/FT4 (+1 SD)	1.07	1.03, 1.11	<0.001	1.02	0.98, 1.07	0.266	1.03	0.99, 1.07	0.158

Model 1: adjusted for age and sex.

Model 2: adjusted for age, sex, smoking, drinking, BMI and NLR.

### Association of sensitivity to THs and MetSSS quartiles

**Table 4** shows the relationship between thyroid hormone sensitivity (per SD increase) and quartiles of MetSSS. The odds ratios for each quartile with 95% confidence intervals, adjusted for age, sex, smoking, drinking, BMI, and NLR, are presented. A significant positive trend was observed across MetSSS quartiles for TSHI and TT4RI (per SD increase), with the strongest association seen in the highest quartile (P for trend < 0.001). TFQI and PTFQI also demonstrated a positive trend, although less pronounced for the second quartile. The FT3/FT4 ratio showed a consistent and significant positive association across all quartiles, suggesting that higher peripheral thyroid hormone sensitivity may be related to a more severe MetS. Individuals in the highest quartile of MetSSS were more likely to have impaired THs sensitivity indices compared to those in the lowest quartile (TFQI: OR=1.16, 95%CI: 1.07-1.21; PTFQI: OR=1.12, 95%CI: 1.06-1.17; TSHI: OR=1.13, 95%CI: 1.06-1.19; TT4RI: OR=1.25, 95%CI: 1.15-1.35; FT3/FT4: OR=1.82, 95%CI: 1.72-1.93).

**Table 4:** Association of sensitivity to thyroid hormones and MetSSS quartiles.

	MetSSS quartiles							P for trend
	Q1	Q2		Q3		Q4		
		OR (95% CI)	P-value	OR (95% CI)	P-value	OR (95% CI)	P-value	
TSHI (+1 SD)	Ref.	1.07 (1.01~1.13)	0.016	1.06 (1.01~1.12)	0.032	1.13 (1.06~1.19)	<0.001	<0.001
TT4RI (+1 SD)	Ref.	1.07 (0.99~1.15)	0.071	1.10 (1.02~1.19)	0.013	1.25 (1.15~1.35)	<0.001	<0.001
TFQI (+1 SD)	Ref.	1.05 (1.00~1.10)	0.053	1.00 (0.96~1.05)	0.935	1.16 (1.07~1.21)	<0.001	<0.001
PTFQI (+1 SD)	Ref.	1.03 (0.98~1.08)	0.231	1.04 (1.00~1.09)	0.082	1.12 (1.06~1.17)	<0.001	<0.001
FT3/FT4 (+1 SD)	Ref.	1.31 (1.24~1.39)	<0.001	1.62 (1.53~1.71)	<0.001	1.82 (1.72~1.93)	<0.001	<0.001

### Exploration of nonlinear relationships

**Figure 2:**
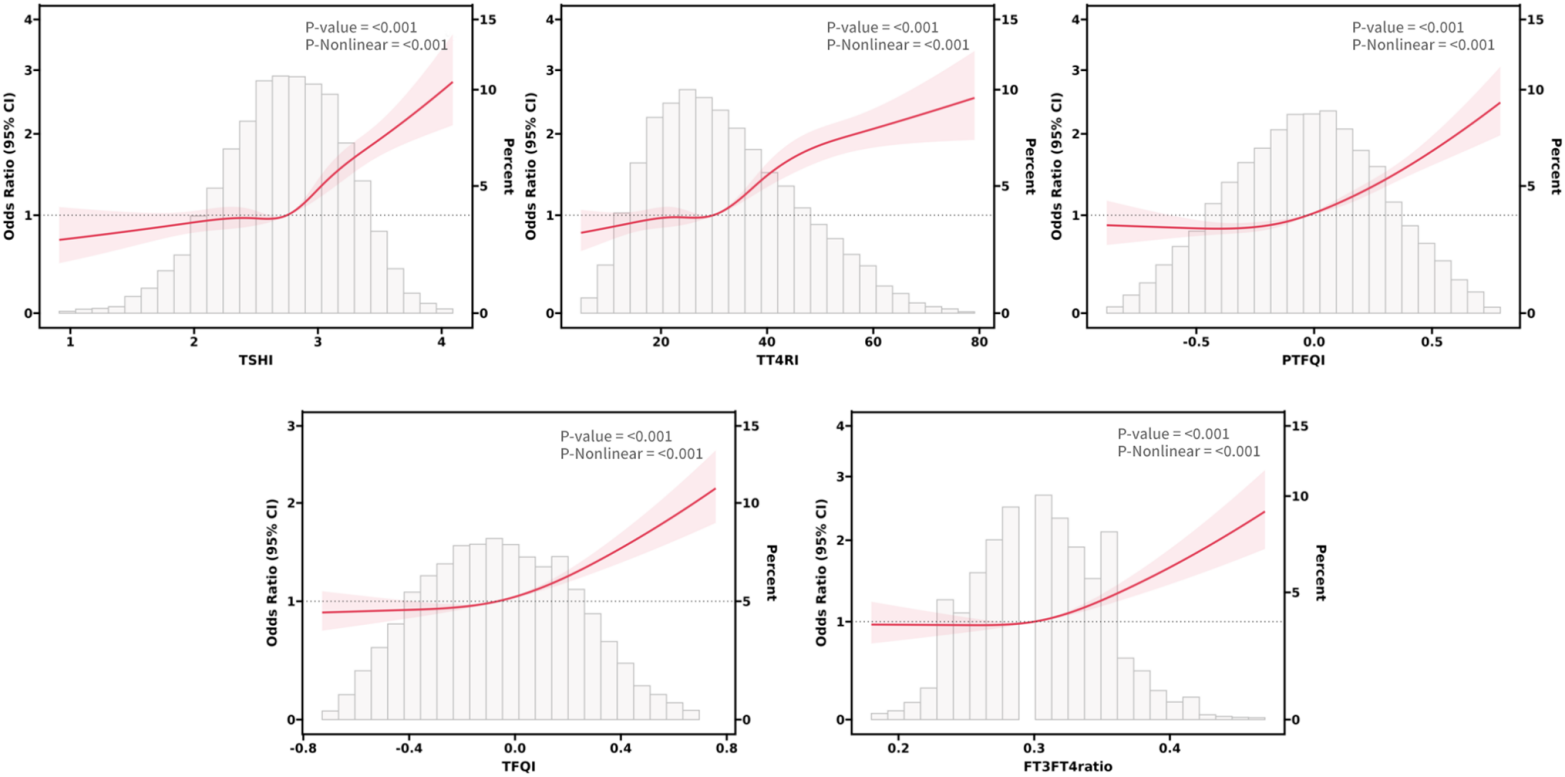
The smooth curve fitting between sensitivity to THs indicators and MetS. Solid red lines and red areas on both sides represent ORs and their 95%CI.

**Figure 3:**
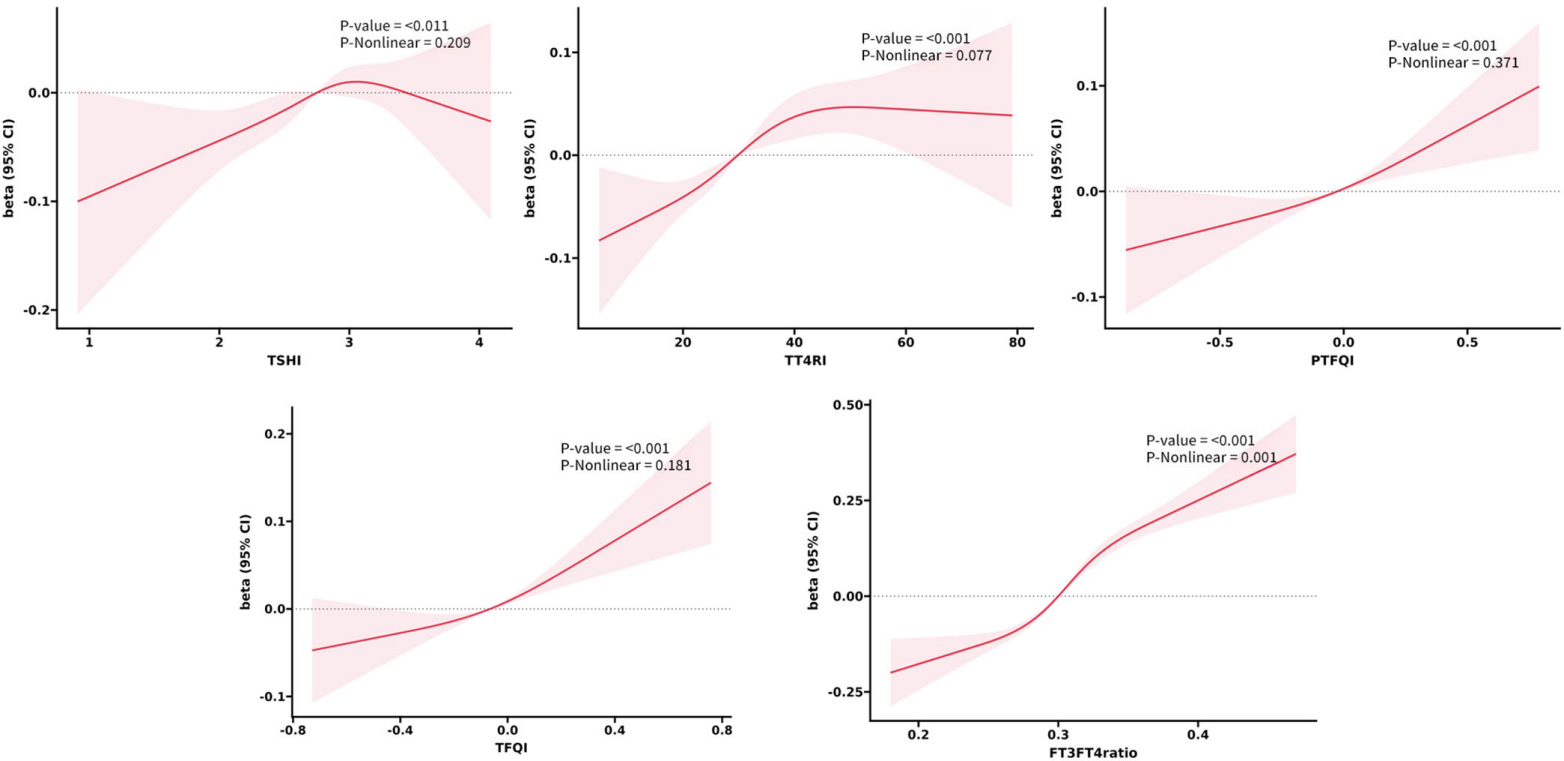
The smooth curve fitting between sensitivity to THs indicators and MetSSS. Solid red lines and red areas on both sides represent the estimated regression coefficient Beta and its 95% confidence interval.

Based on the results of regression analysis, we conducted a restricted cubic spline analysis to investigate the dose-response relationship between indicators of THs sensitivity and MetS (**Figure 2**). After adjusting for potential confounders such as age, sex, smoking, drinking, and NLR, we found a significant association between sensitivity to thyroid hormones indices and MetS in all euthyroid participants (overall P<0.001). Non-linear relationships were observed between all indicators of thyroid hormone sensitivity and MetS (P for nonlinearity<0.001). Furthermore, both central and peripheral sensitivity to thyroid hormones were positively correlated with MetSSS) (overall P<0.05 (**Figure 3**). TSHI, TT4RI, TFQI, and PTFQI showed a linear association with MetSSS (P for nonlinearity>0.05), while FT3/FT4 exhibited a non-linear relationship with MetSSS (P for nonlinearity=0.001).

### Subgroup analysis

**Figure 4:**
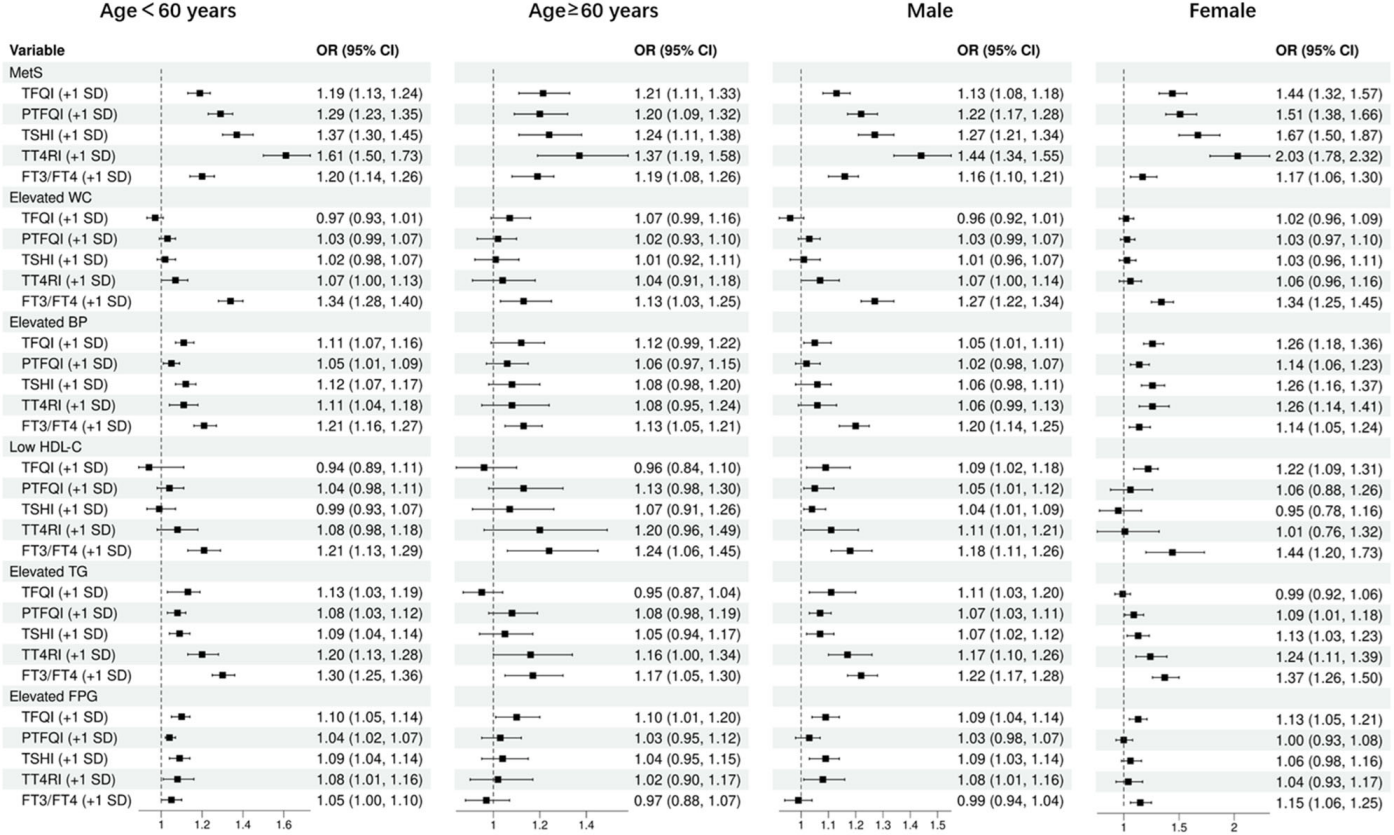
Subgroup analysis by age and gender.

As shown in **Figure 4**, after adjusting for potential confounders, the odds ratios (ORs) for the risk of MetS were found to increase with every 1 standard deviation increase in TFQI, PTFQI, TSHI, TT4RI, and FT3/FT4 ratio. These associations were observed in individuals under 60 years old and those over 60 years old, as well as in males and females. specifically, the ORs for MetS risk were 1.19 to 1.61 among individuals under 60 years old, 1.21 to 1.37 among those over 60 years old, 1.13 to 1.44 in males, and 1.44 to 2.03 in females. Additionally, the FT3/FT4 ratio was positively associated with all MetS components risk in all subgroups. however, most central sensitivities to THs were not significantly associated with elevated WC and low HDL-C. They were positively associated with elevated blood pressure (BP) and triglycerides (TG) in females and individuals under 60 years old, and all sensitivity to THs were positively associated with elevated FPG in subjects under 60 years old.

## Discussion

To the best of our knowledge, this is the first large-sample cross-sectional study to evaluate the association between central and peripheral thyroid hormones sensitivity and risk of MetS and MetSSS in euthyroid Chinese adults. The results provide evidence that both decreased central sensitivity to THs (elevated TFQI, PTFQI, TSHI, and TT4RI) and increased peripheral sensitivity to THs (elevated FT3/FT4) were associated with an increased risk of MetS and MetSSS in euthyroid Chinese adults. Additionally, subgroup analysis showed that these relationships remain stable regardless of sex and age. This analysis goes beyond simply examining the absolute levels of FT3, FT4, and TSH, and provides insight into the resistance of THs within MetS and MetSSS.

THs have been found to play a significant role in all aspects of MetS through various mechanisms. These hormones can impact metabolic rate, appetite control, and sympathetic activity, which in turn affects adiposity (33). The stimulation of the sympathetic nervous system by THs also influences glucose and lipid metabolism, as well as cardiovascular regulation (34). However, fluctuations in THs levels within both normal and abnormal ranges have been linked to metabolic disorders. Previous studies have shown that levels of TSH or THs alone may not fully explain the relationship between the thyroid system and metabolic dysfunction (35). Therefore, comprehensive indices that reflect THs homeostasis may provide a more accurate understanding of this relationship. Laclaustra et al. have proposed the use of TFQI and PTFQI to quantify the relationship between thyroid function and metabolic indices (16). This approach, based on the theory of THs resistance, aims to explain contradictory findings in research and has opened up new avenues for studying the connection between thyroid function and metabolic disorders.

Numerous studies have investigated the relationship between sensitivity to THs and metabolic disorders. A recent study involving 31,678 patients with coronary heart disease found that impaired sensitivity to THs was significantly linked to dyslipidemia, regardless of gender, glucose levels, and blood pressure (35). Another recent cross-sectional study also showed a positive correlation between TFQI and FT3/FT4 levels with lipid levels (36). Our study revealed that all measures of THs sensitivity were positively linked to low HDL-C and high TG levels in euthyroid individuals. Research has demonstrated that TSH directly influences the expression of HMG-CoA reductase in the liver, leading to increased cholesterol synthesis (37, 38). TSH binds to its receptor and regulates protein kinase activation through the cyclic adenosine monophosphate/protein kinase A pathway, altering its inhibitory effect on the peroxisome proliferator-activated receptor-γ signaling pathway. This, in turn, triggers the activity of sterol regulatory element-binding protein (SREBP) 1c in the liver, promoting the expression of genes related to fat formation (39). SREBP is a transcription factor that positively regulates the expression of LDLR and cholesterol synthesis, with T3 mediating cholesterol synthesis through SREBP-1 and SREBP-2 (40, 41). These established mechanisms illustrate the close connection between THs sensitivity and dyslipidemia. Another study conducted on a cross-sectional basis found that the TFQI was linked to a higher prevalence of diabetes in both 2296 euthyroid adults in America and 8319 euthyroid adults in China (42). It has been noted that TSH can stimulate the secretion of leptin in human adipose tissue, which in turn reduces insulin secretion and synthesis in pancreatic β-cells. Therefore, the combined index TFQI, which takes into account both TSH and FT3, may offer a more accurate indication of the risk of developing diabetes compared to individual indices in individuals with normal TSH, FT3, and FT4 levels.

Furthermore, several studies have suggested that THs can directly and indirectly influence blood pressure. Insensitivity to THs can result in a decrease in the dilation of arterial smooth muscle, availability of nitric oxide, and endothelium-dependent vasodilation (43–45). A recent study also found a positive association between decreased central sensitivity to THs and visceral adipose tissue (46), which aligns with our findings. Disorders in THs sensitivity can disrupt the body’s metabolic balance, affecting the functioning of adipose tissue. While THs typically stimulate both brown and white adipose tissue, promoting thermogenesis and energy expenditure, impaired thyroid hormone sensitivity can lead to reduced activity in brown adipose tissue and impaired browning of white adipose tissue, resulting in decreased energy expenditure and a tendency for fat accumulation, particularly in the abdominal region (47, 48).

Previous studies examining the relationship between the FT3/FT4 ratio and MetS have produced conflicting results. A recent cross-sectional study found that lower FT3/FT4 levels were linked to MetS components,^42^ contradicting our findings. Greet et al. discovered a positive association between FT3, FT4, and the FT3/FT4 ratio with MetS components (49). Shon et al. observed a negative correlation between FT4 levels within the normal range and BMI (50). Roos et al. reported associations between low normal FT4 levels and increased insulin resistance and an unfavorable lipid profile in euthyroid adults (51). In a study involving 3,148 subjects, it was found that higher levels of FT4 were associated with higher levels of HDL cholesterol and lower levels of waist circumference, insulin resistance, and insulin (52). Additionally, previous studies have shown that higher levels of fT3 are linked to increased body fat mass and various metabolic parameters. A higher ratio of fT3 to fT4 has been associated with a less favorable metabolic profile and increased placental growth in pregnant women (53). Research has suggested that individuals with higher fat mass and/or less favorable metabolic profiles may have increased activity of deiodinase (DIO) 1 and/or 2, leading to higher conversion of fT4 to fT3 (54). This enhanced sensitivity to thyroid hormones can impact metabolic rate, glucose absorption and utilization, and lipid metabolism, potentially leading to fluctuations in blood sugar and lipid levels (55). It may also worsen insulin resistance, affect insulin signaling pathways, and increase cardiovascular activity, all of which are characteristics of metabolic syndrome. Essentially, an excessive response to normal thyroid hormone levels may increase the risk of developing metabolic abnormalities associated with MetS.

To further elaborate on the intricate relationship between thyroid function and metabolic regulation, it is essential to consider the role of circadian rhythms. Emerging evidence underscores the bidirectional interplay between thyroid hormones and circadian clocks (56). The hypothalamus-pituitary-thyroid axis demonstrates circadian oscillation, with thyroid hormone secretion orchestrated by both central and peripheral clocks. In turn, thyroid hormones modulate clock gene rhythmicity. Notably, triiodothyronine (T3) acts as a temporal cue for the central circadian clock by enhancing Bmal1 promoter activity, ensuring optimal metabolic regulation (57). Conversely, circadian disruptions, such as those in hypothyroidism, alter suprachiasmatic nucleus clock gene expression and impact metabolic parameters like oxygen consumption and body temperature, potentially contributing to metabolic syndrome (58). Importantly, aligning circadian rhythms through strategies like time-restricted feeding can restore metabolic health and enhance thyroid hormone signaling, underscoring the importance of circadian integrity in thyroid-metabolism axis regulation (59, 60). This interplay between circadian rhythms and thyroid function provides a more comprehensive framework for understanding metabolic regulation and suggests potential therapeutic avenues for addressing metabolic disorders.

The clinical importance of our study’s results lies in its potential to improve the understanding and management of MetS in euthyroid individuals. By identifying the association between thyroid hormone sensitivity and MetS risk, clinicians can consider evaluating thyroid function more comprehensively using indices like TFQI and FT3/FT4 ratio, rather than relying solely on traditional markers such as TSH, FT3, and FT4. This approach may allow for earlier identification of individuals at risk of MetS, enabling timely interventions to prevent or delay the onset of metabolic complications. Additionally, the findings highlight the need for further research into the therapeutic potential of modulating thyroid hormone sensitivity as a strategy for MetS management.

There were several limitations that should be acknowledged. Firstly, the cross-sectional design restricts the ability to establish a cause-and-effect relationship between thyroid hormone sensitivity indices and MetS. Additionally, the generalizability of the findings may be limited as the study was conducted in a Chinese population and may not be applicable to other ethnicities or regions. Lastly, the study did not consider all potential confounding factors, such as diet patterns or physical activity levels, that could impact the relationship between TH sensitivity and MetS.

## Conclusion

In summary, this study presents evidence of a significant correlation between impaired thyroid hormone sensitivity and MetS, along with its severity, in euthyroid adults. The relationships with waist circumference, dyslipidemia, hyperglycemia, and hypertension emphasize the possible involvement of thyroid hormones in the pathogenesis of MetS. Future investigations should take into account thyroid hormone sensitivity indices when assessing MetS risk and further explore the mechanisms that drive these associations.

## Data Availability

The raw data supporting the conclusions of this article will be made available by the authors, without undue reservation.
